# Transcriptomic and Metabolomic Analyses of the Mechanisms Underlying Zearalenone Removal by *Lactobacillus paracasei*

**DOI:** 10.3390/toxins18090382

**Published:** 2026-09-04

**Authors:** Min Gan, Peirong Chen, Rui Zeng, Fuhua Wang, Yarong Zhao

**Affiliations:** 1 Institute of Quality Standard and Monitoring Technology for Agro-Products of Guangdong Academy of Agricultural Sciences, Guangzhou 510640, China; 2 Guangdong Engineering Laboratory of Biomass Value-Added Utilization, Institute of Biological and Medical Engineering, Guangdong Academy of Sciences, Guangzhou 510316, China; 3 Guangdong Association of Agricultural Standardization, Guangzhou 510640, China

**Keywords:** *Lactobacillus paracasei*, zearalenone, transcriptome, metabolome, carbohydrate metabolism

## Abstract

Zearalenone (ZEN) is a mycotoxin produced by Fusarium species that is harmful to agricultural crops. Therefore, the prevention and control of ZEN contamination is an important concern for food safety. *Lactobacillus paracasei* 85 is a well-recognized probiotic with considerable potential for mycotoxin biocontrol owing to its diverse probiotic properties and antimicrobial activity. Based on its reported potential, we aimed to evaluate its ability to degrade ZEN using degradation assays under optimal conditions. Transcriptome sequencing, untargeted metabolomics, and integrated bioinformatics analyses were conducted to explore the molecular mechanisms underlying ZEN removal. Transcriptomic analysis showed that the strain activated central carbon and secondary metabolism during ZEN removal, and all seven differentially expressed genes closely related to carbohydrate metabolism were downregulated. Metabolomic analysis revealed that lipid and amino acid metabolism, in addition to carbohydrate metabolism, played important roles in ZEN removal. Correlation analysis between carbohydrate metabolism-related genes and their metabolites revealed that these genes were significantly associated with four metabolites: myo-inositol, guanosine, glycolic acid, and trehalose. As key nodes in the carbohydrate metabolism pathway, changes in the expression of these four metabolites and their interactions with genes may represent key regulatory factors responsible for the overall downregulation of carbohydrate metabolism.

## 1. Introduction

Zearalenone (ZEN), also known as the F-2 toxin, is a secondary metabolite synthesized by *Fusarium graminearum* and other phytopathogenic fungi of the Fusarium genus. As a prototypical nonsteroidal estrogenic mycotoxin, ZEN predominantly contaminates agricultural crops and their derivatives, including corn, barley, wheat, and sorghum [1,2]. Due to its structural similarity to endogenous estrogens, ZEN has the potential to disrupt the endocrine system and trigger severe health threats to human and animal health, including reproductive toxicity, gastrointestinal toxicity, hepatotoxicity, and immunotoxicity [3,4,5]. The International Agency for Research on Cancer has classified ZEN as a Group 3 carcinogen. Multiple countries and regions have formulated stringent maximum residue limits for ZEN in cereals and cereal products, including China, France, Russia, Uruguay and the European Union [6,7,8]. ZEN contamination is a growing global concern that is exacerbated by climate change. Surveys indicate that 84% of grain samples in India are contaminated with ZEN, 33% of which exceed the EU’s maximum permissible limit [9,10,11]. In the United States, approximately 10 million tons of corn and wheat flour are estimated to be contaminated with ZEN annually [12]. A large-scale survey covering 3420 samples from China and South Korea reported a ZEN detection rate of 47%, with Chinese corn samples presenting an average ZEN concentration of 494 μg/kg and a maximum detected level up to 10,374 μg/kg [13].

Currently, the primary strategies for ZEN removal are physical, chemical, and biological methods. Because of the high thermal stability of ZEN, conventional treatment methods often exhibit limited removal efficiency [14,15]. Compared to conventional detoxification strategies, biological removal has garnered significant attention and widespread application owing to its environmental friendliness, high efficiency, and lack of toxic residues [16,17]. In our previous study, a lactic acid bacterium was isolated from 20 samples of naturally fermented pickle brine collected from rural households in Southwest China. Based on morphological identification and 16S rDNA sequence analysis, the strain was identified as *Lactobacillus paracasei* and was designated as *L. paracasei* 85. *L. paracasei* is a well-recognized probiotic with considerable potential for mycotoxin biocontrol owing to its diverse probiotic properties and antimicrobial activity. Our previous study indicated that the strain achieved a degradation rate of 78.4% at a concentration of 5.0 μg/mL ZEN [18]. Notably, most related studies only focus on phenotypic verification of ZEN removal capacity of probiotic strains, while the underlying molecular mechanisms remain ambiguous, which limits the in-depth application and industrial promotion of detoxification strains. Therefore, this study aimed to integrate transcriptomic and metabolomic analyses to systematically elucidate the biological processes underlying the ZEN removal capacity of *L. paracasei* 85.

This study employed integrated transcriptomic and metabolomic analyses to overcome the limitation of traditional analyses and enrich the mechanistic basis of probiotic detoxification. It has systematically explored the key biological pathways, core functional genes, and differential metabolites dominating ZEN elimination by *L. paracasei* 85. Distinct from conventional phenotype-oriented studies, our multi-omics experimental data clearly clarified the molecular reprogramming characteristics of central carbon metabolism, carbohydrate metabolism, lipid metabolism, and amino acid metabolism during ZEN stress responses and detoxification.

## 2. Results

### 2.1. Quality Control and Sequence Alignment Analysis of Transcriptome Sequencing Data

The sequencing data for the samples are presented in Table 1. Initially, raw reads were filtered to eliminate adapter sequences and sequences with excessively ambiguous bases, resulting in clean reads. The quality of the sequencing data was assessed by analyzing the base composition and quality distributions. Following quality control procedures, the average raw paired-end reads for the *L. paracasei* 85 samples amounted to 3.12 G, with an average number of valid paired-end reads of 3.02 G. The percentages of bases with Phred scores greater than 20 and 30 were 98.23% and 95.06%, respectively. The average number of mapped reads against the reference genome was 2.83 G, with an average genome mapping rate of 93.71%. These findings indicate that the RNA sequencing data were of high quality and appropriate for subsequent bioinformatics analyses.

### 2.2. Screening of Differentially Expressed Genes

**Figure 1:**
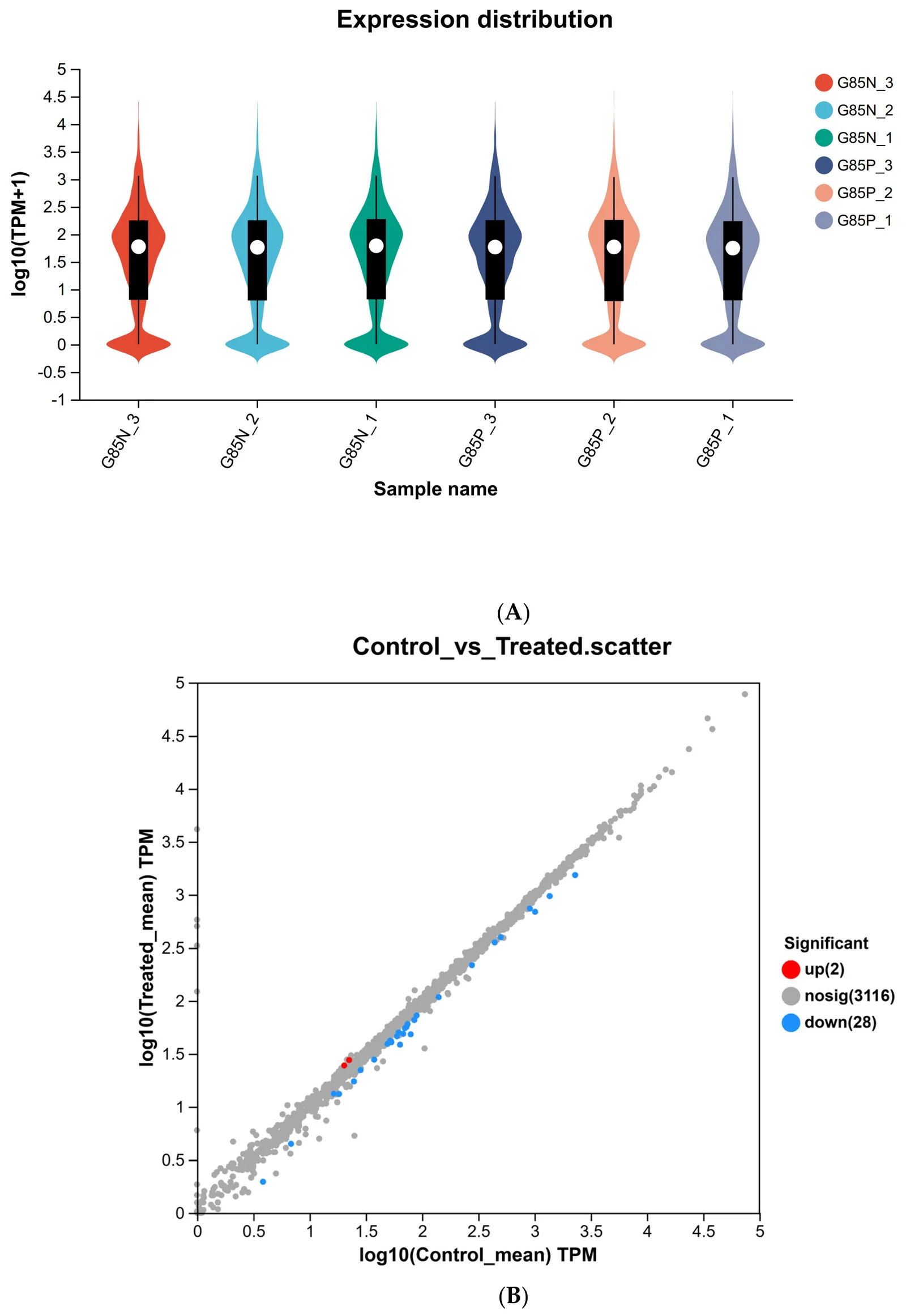
Density distribution of gene expression (**A**) and scatter plot of differentially expressed genes (**B**) in *Lactobacillus paracasei* 85 following Zearalenone (ZEN) treatment.

A Venn analysis of the gene expression profiles between the experimental and control groups revealed the expression of 2550 and 2540 genes in the respective groups. Of these, 2536 genes were co-expressed, whereas 14 and 4 genes were uniquely expressed in the experimental and control groups, respectively, indicating a limited number of specifically expressed genes under co-culture conditions. Differential expression analysis was conducted using the read counts to identify genes with significantly different expression. The gene expression density distribution demonstrated high consistency across biological replicates in the experimental group, indicating robust reproducibility of the experiment (Figure 1A). The DESeq2 algorithm was used for differential expression analysis. Using screening thresholds of ∣log_2_(fold change)∣ > 1.2 and *p* < 0.05, 30 differentially expressed genes were identified. Compared with the control group, two genes were upregulated and 28 genes were downregulated (Figure 1B).

### 2.3. Gene Ontology (GO) Annotation and Enrichment of Differentially Expressed Genes

**Figure 2:**
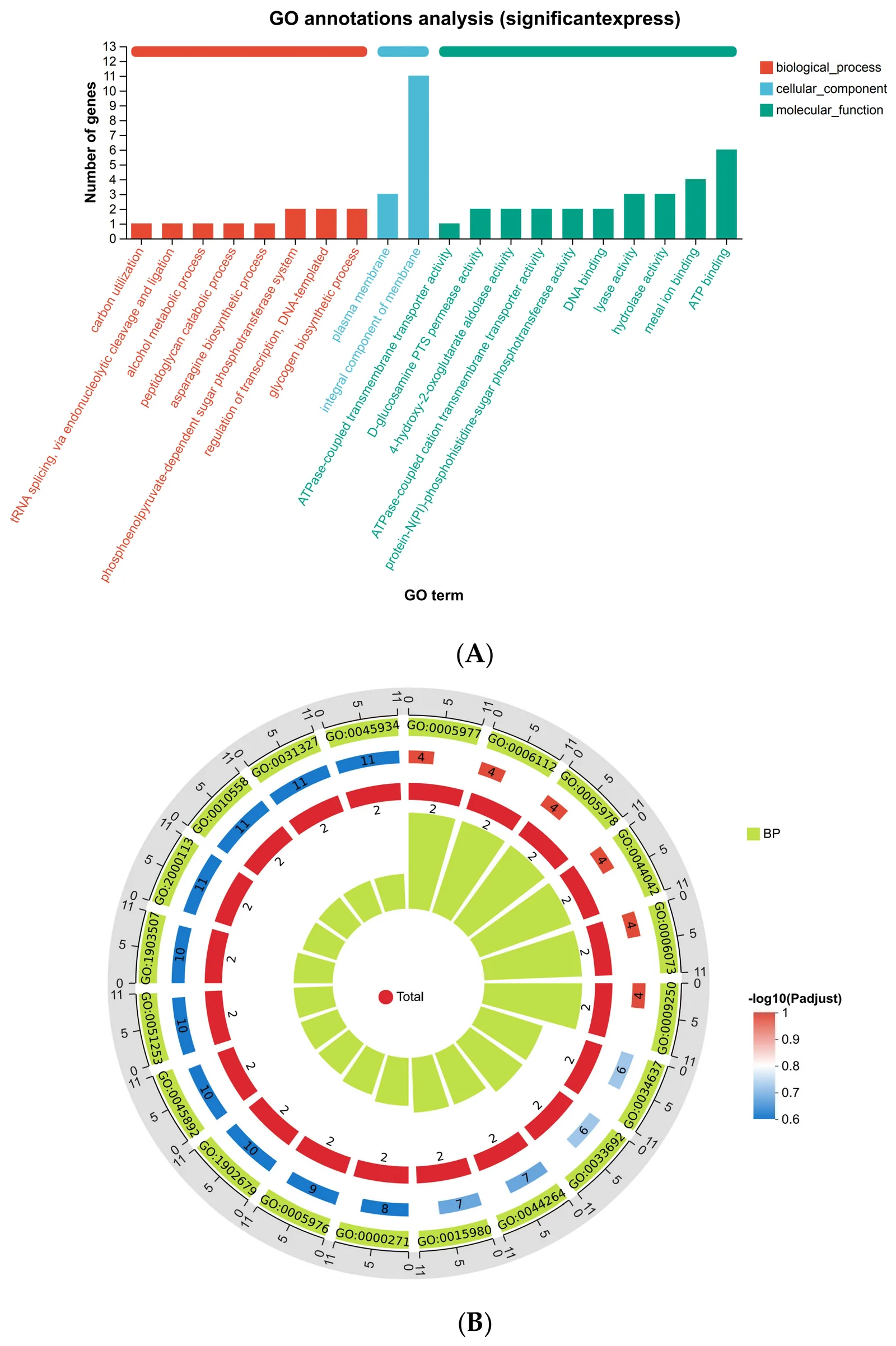
Gene Ontology (GO) annotation (**A**) and enrichment circle diagram (**B**) for differentially expressed genes in *L. paracasei* 85 after ZEN treatment.

According to the GO system, gene functions were categorized into three major categories: Molecular Function (MF), Cellular Component (CC), and Biological Process (BP). The annotation results categorized all 30 differentially expressed genes into three distinct groups during ZEN elimination by *L. paracasei* 85. Within the BP category, the predominant terms encompassed the glycogen biosynthetic process, phosphoenolpyruvate-dependent sugar phosphotransferase system (PTS), and DNA-templated regulation of transcription. In the CC category, genes were primarily associated with integral components of the membrane and plasma membrane. In the MF category, the principal terms included ATP binding, metal ion binding, hydrolase activity, and lyase activity (Figure 2A). GO functional enrichment analysis revealed that the differentially expressed genes were significantly enriched in 20 GO terms. The most highly enriched GO terms were predominantly related to biological processes, such as carbon utilization, asparagine biosynthetic processes, 4-hydroxy-2-oxoglutarate aldolase activity, glucan biosynthetic and metabolic processes, and glycogen biosynthetic and metabolic processes (Figure 2B).

### 2.4. Kyoto Encyclopedia of Genes and Genomes (KEGG) Annotation and Enrichment of Differentially Expressed Genes

**Figure 3:**
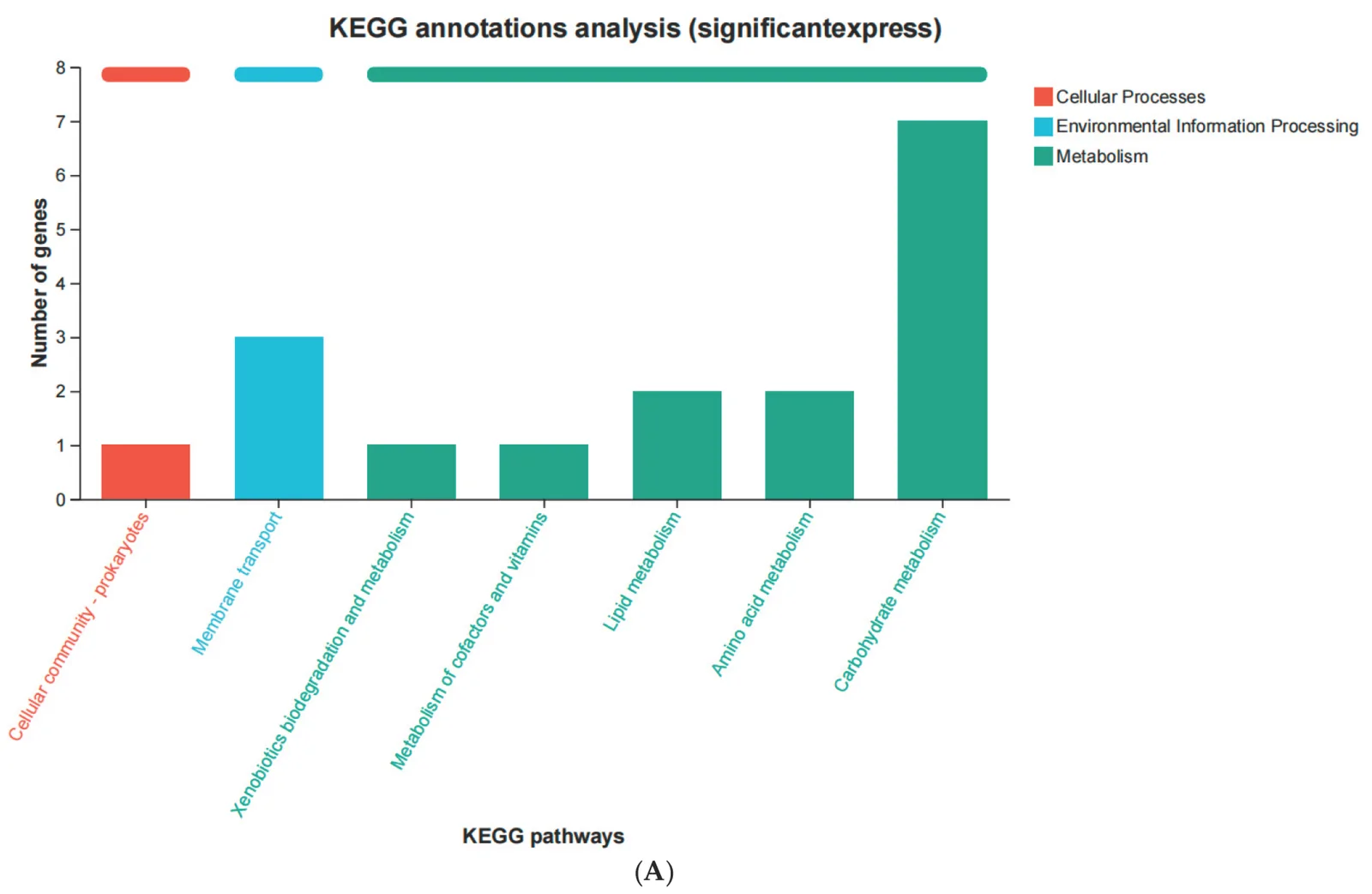

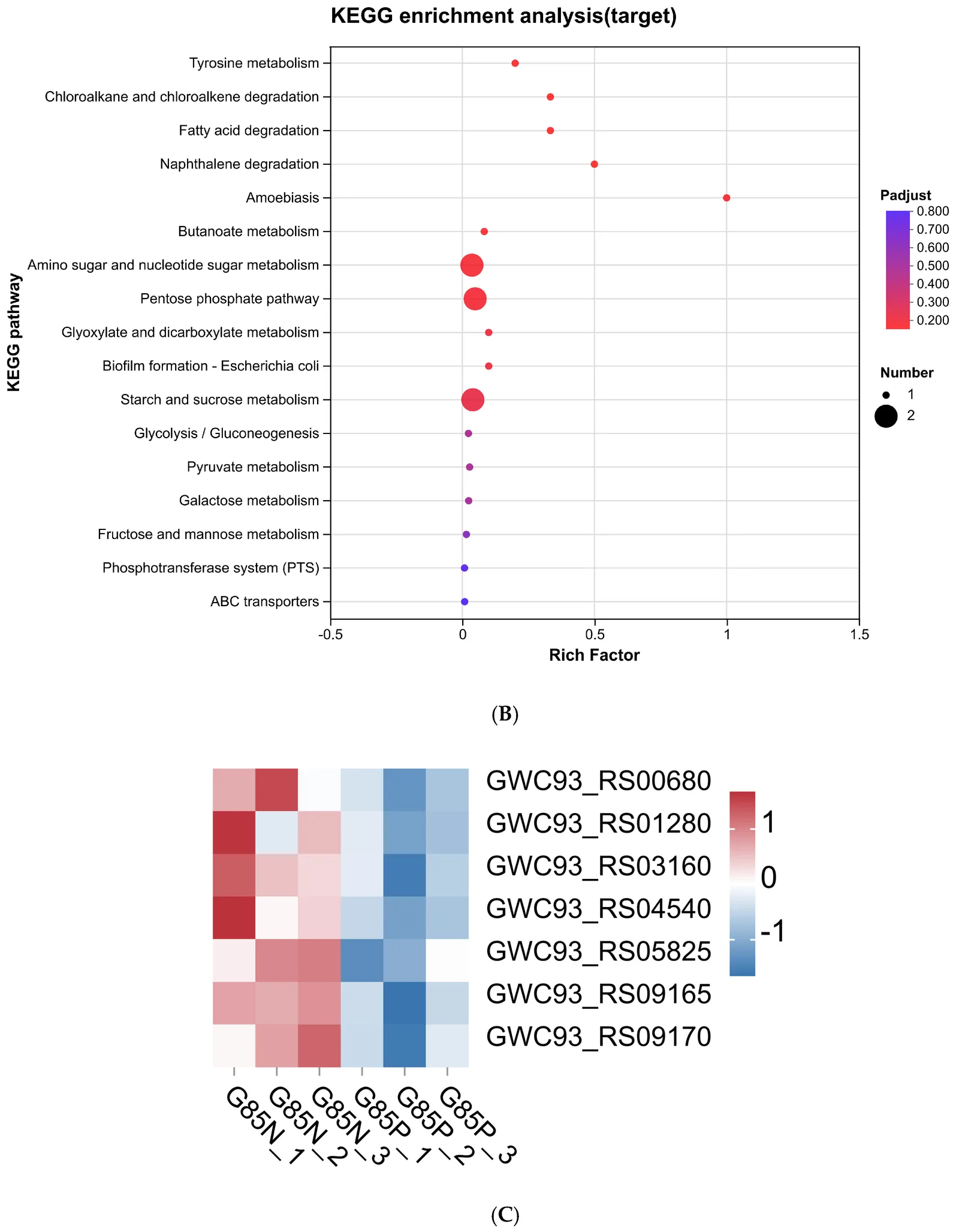
GO annotations (**A**), enrichment circle diagram (**B**) and heat map of target genes. (**C**) Differentially expressed genes involved in sugar metabolism in *L. paracasei* 85 following ZEN treatment.

Genes were categorized into various pathways based on their biological functions using the KEGG database. As shown in Figure 3A, three primary pathway categories were identified for ZEN removal by *L. paracasei* 85: Metabolism, Environmental Information Processing, and Cellular Processes. Within the metabolic category, the pathways included amino acid, carbohydrate, lipid, and cofactor and vitamin metabolism, and xenobiotic biodegradation and metabolism. Enrichment analysis revealed that 30 differentially expressed genes were significantly associated with 17 pathways. The pathways for amino sugar and nucleotide sugar metabolism, the pentose phosphate pathway, and starch and sucrose metabolism were significantly enriched (Figure 3B). The pentose phosphate pathway primarily produces substantial quantities of NADPH. As a crucial cofactor, NADPH is vital for maintaining cellular reducing power and plays a role in the detoxification of endogenous and exogenous oxidants and electrophiles [19]. The pronounced enrichment of genes in this pathway suggests that the strain may augment this pathway to provide adequate reducing power for detoxification processes.

All cells are encapsulated by lipid membranes that facilitate interactions between the cells and their environment. The intricate composition of membrane lipids determines the structural properties and functions of these membranes [20]. In this study, the genes that were differentially expressed and enriched in lipid metabolism were upregulated. Under toxin-induced stress, this strain may modify its cell membrane structure through enhanced lipid synthesis and metabolism, thereby improving its resistance to stress. Cofactors and vitamins are integral to a broad spectrum of cellular and physiological processes. For example, vitamin B6 serves as a crucial cofactor in numerous biochemical reactions and regulates fundamental cellular metabolism, thereby influencing overall physiological function [21]. Transcriptomic analysis revealed the upregulation of genes associated with cofactor and vitamin metabolism, suggesting that the strain maintained a highly active physiological state to counteract adverse stress conditions. Subsequently, seven differentially expressed genes closely linked to carbohydrate metabolism were identified from the transcriptomic data. These genes were annotated as glucose-1-phosphate adenylyltransferase (GWC93_RS09170), 1,4-α-glucan branching enzyme GlgB (GWC93_RS09165), bifunctional aldolase HgaA/KdgA (GWC93_RS04540), bifunctional acetaldehyde-CoA/alcohol dehydrogenase (GWC93_RS00680), PTS sorbitol transport subunit IIB (GWC93_RS05825), ribokinase (GWC93_RS03160), and PTS glucitol/sorbitol transport protein subunit IIC (GWC93_RS05820).

The selected target genes for expression pattern analysis are depicted in Figure 3C; all seven genes were downregulated. This observation suggests that the strain extensively modulates central carbon and secondary metabolism during ZEN removal, thereby providing the precursors and energy required for detoxification.

### 2.5. Real-Time Quantitative PCR (RT-qPCR) Validation of Differentially Expressed Genes Related to Carbohydrate Metabolism

**Figure 4:**
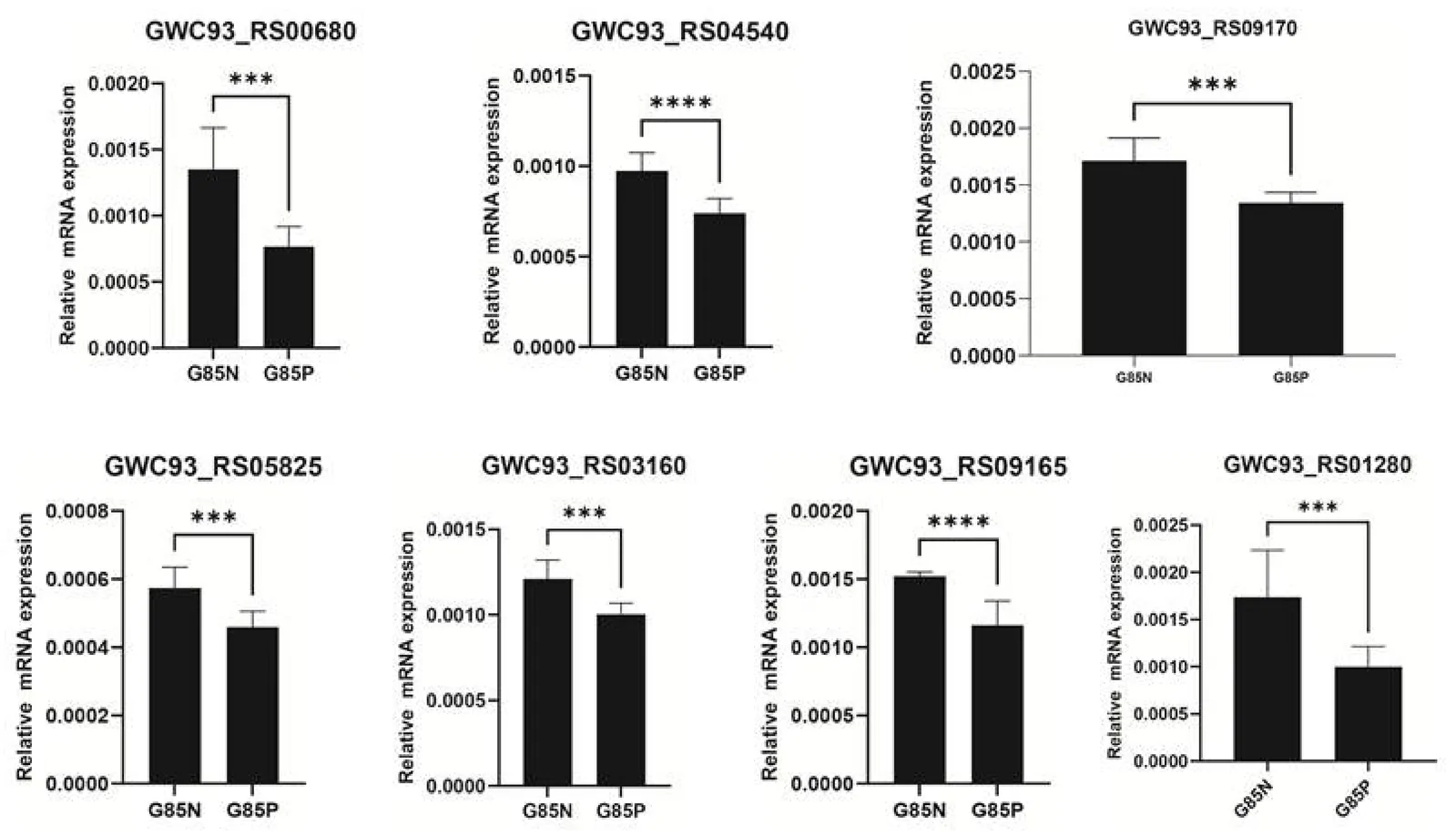
Real-time quantitative PCR (RT-qPCR) validation of RNA-Seq transcriptome data. “***” means *p* < 0.001 and “****”means *p* < 0.0001 in the figures.

To validate the reliability of the transcriptomic data, RT-qPCR was conducted on the seven differentially expressed genes associated with carbohydrate metabolism using NR_025880.1 as the reference gene. The gene expression levels were normalized, and the fold changes were transformed into log_2_ values. As illustrated in Figure 4, the expression patterns of these genes, as determined by RT-qPCR, were highly consistent with those obtained from transcriptome sequencing, thereby corroborating the credibility of the transcriptomic analysis.

### 2.6. Metabolomic Data Analyses

**Figure 5:**
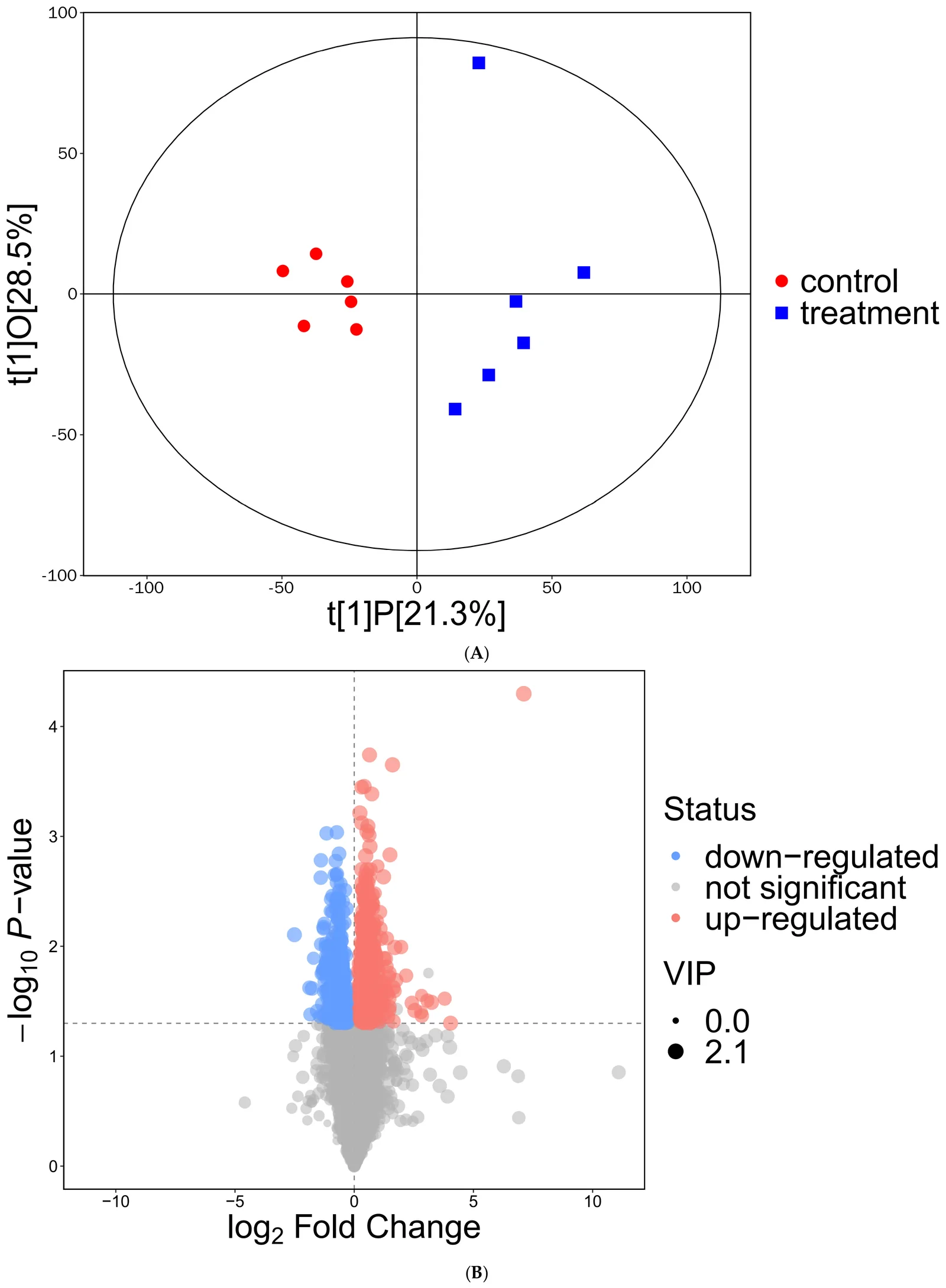

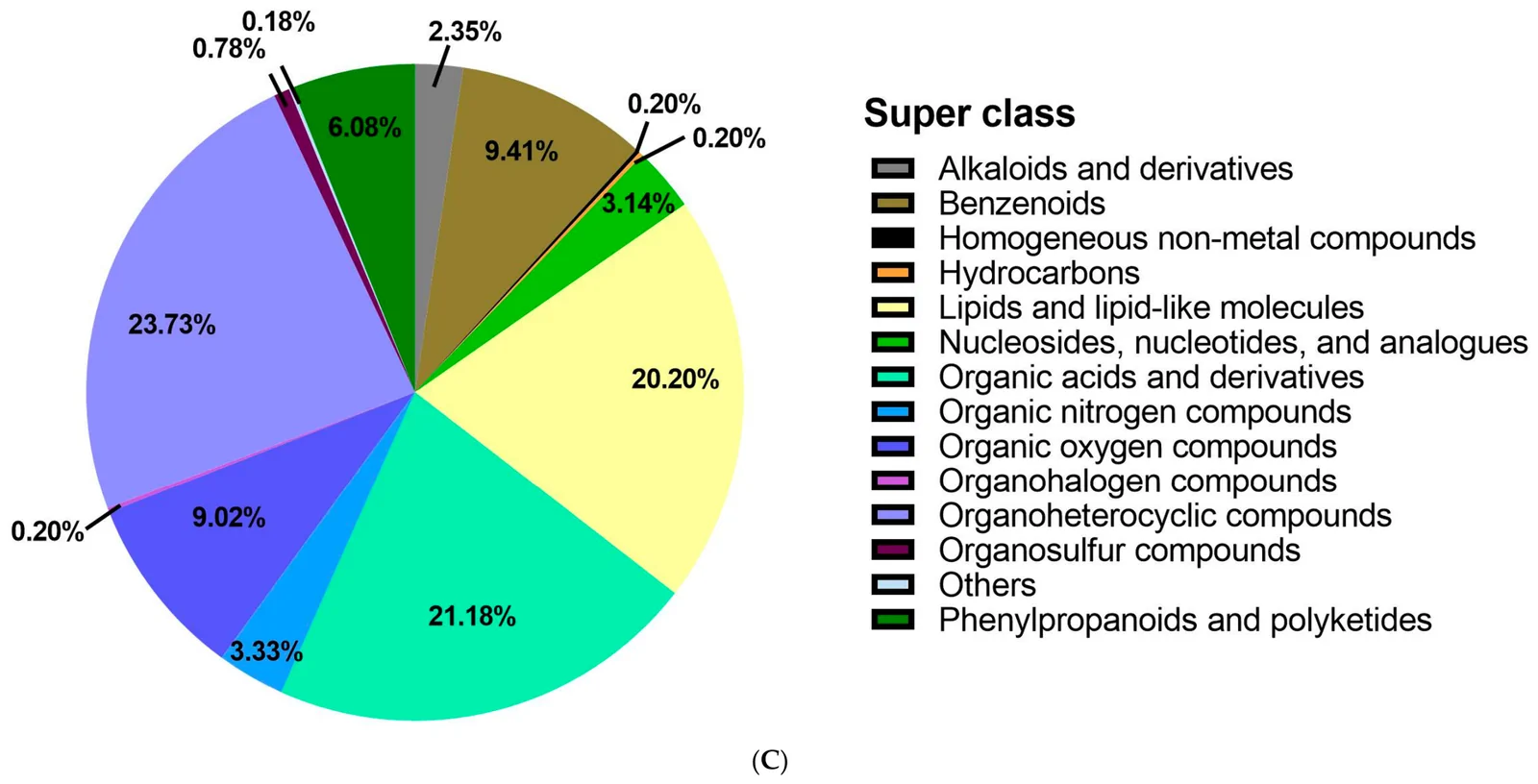
Orthogonal partial least squares-discriminant analysis (**A**), volcano plot for screening differential metabolites (**B**) and pie chart of metabolite classification and proportion (**C**) in *L. paracasei* 85 following ZEN treatment.

Orthogonal partial least square-discriminant analysis is a supervised multivariate statistical technique designed to eliminate orthogonal variables that are irrelevant to grouping factors, thereby allowing for the separate analysis of non-orthogonal and orthogonal variables. This approach enhances the reliability of detecting differences between the groups. As illustrated in Figure 5A, all sample points fell within the 95% confidence interval, and the experimental and control groups were clearly distinguished. This separation indicated that the data were suitable for subsequent screening of differential metabolites. Differential metabolites were identified using the criteria of a variable importance in projection score > 1 and a *p*-value < 0.05, and the results were visualized using volcano plots. A total of 1081 differential metabolites were identified, comprising 565 upregulated and 516 downregulated metabolites. These findings indicate that ZEN significantly influences the intracellular metabolism of *L. paracasei* 85. As depicted in Figure 5C, the significantly different metabolites were predominantly categorized into organoheterocyclic compounds, organic acids and derivatives, and lipids and lipid-like molecules, accounting for 23.725%, 21.176%, and 20.196% of the total metabolites, respectively.

### 2.7. Analysis of Differential Metabolites

**Figure 6:**
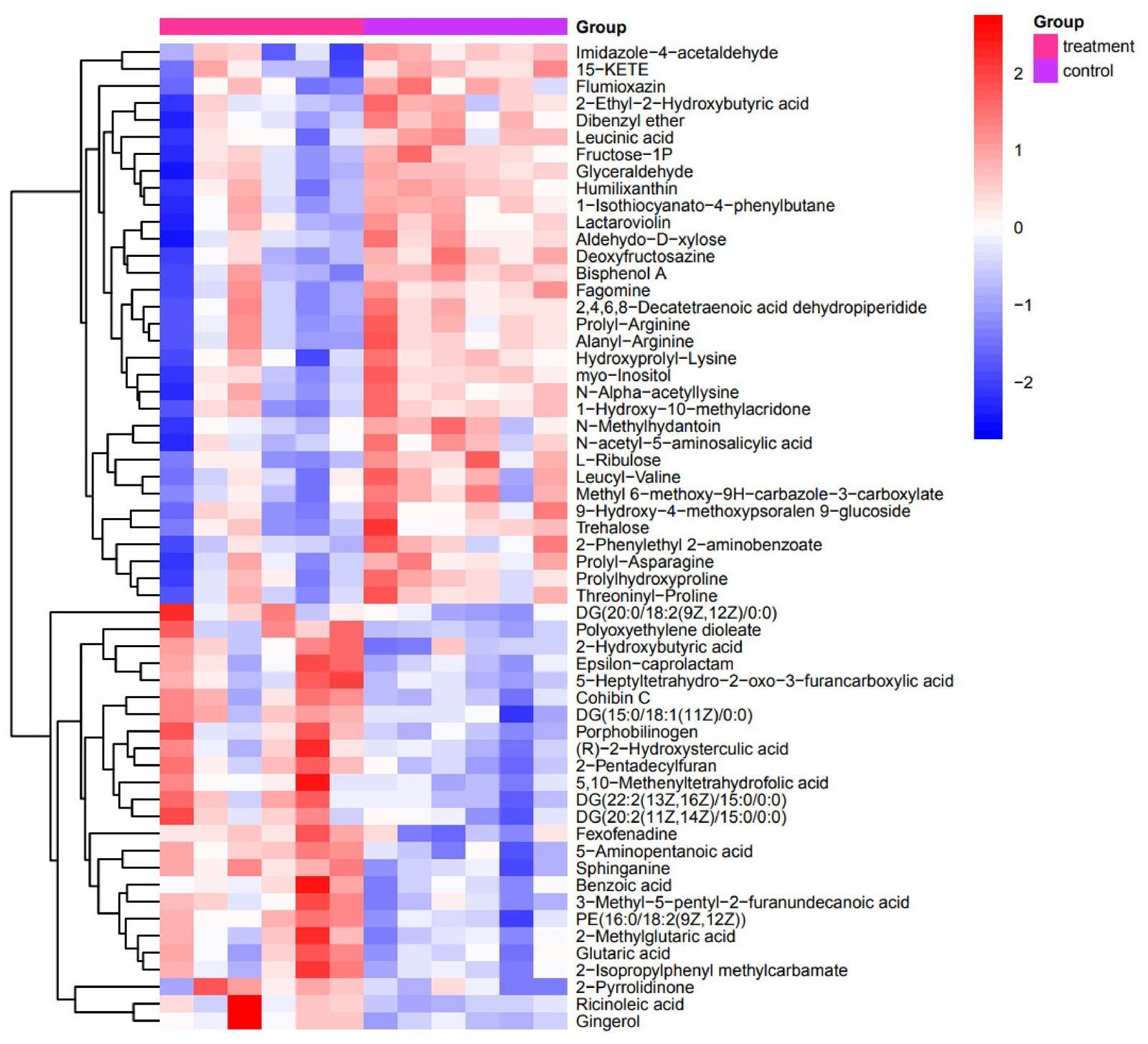
Hierarchical cluster analysis of differential metabolites in *L. paracasei* 85.

As shown in Figure 6, ricinoleic acid, diacylglycerol (DG(20:0/18:2(9Z,12Z)/0:0)), and 5-aminopentanoic acid exhibited elevated expression. Ricinoleic acid, a long-chain unsaturated fatty acid, is a metabolite of oleic acid that primarily participates in lipid metabolism in organisms [22]. DG(20:0/18:2(9Z,12Z)/0:0) is a diacylglycerol whose synthesis begins with glycerol-3-phosphate, which is predominantly derived from dihydroxyacetone phosphate. Glycerol-3-phosphate is initially acylated by acyl-CoA to form lysophosphatidic acid. This intermediate is subsequently acylated by an additional acyl-CoA molecule to produce phosphatidic acid. Phosphatidic acid dephosphorylation subsequently leads to the formation of diacylglycerols. In parallel, 5-aminopentanoic acid, a product of lysine degradation, is synthesized endogenously or generated through bacterial lysine catabolism and serves as an intermediate in the lysine degradation pathway. Within this pathway, lysine is catabolized to succinate, glutarate, and acetyl-CoA. The marked upregulation of these metabolites indicates that lipid and amino acid metabolism are crucial for the elimination of ZEN. Conversely, reduced expression of trehalose, 9-hydroxy-4-methoxypsoralen, 9-glucoside, and deoxyfructosazine were observed. In bacteria capable of using starch, glycogen, or maltodextrin as substrates, trehalose synthase catalyzes the conversion of maltose to trehalose through the isomerization of the α-1,4 glycosidic bond of maltose into an α,α-1,1′-glycosidic bond [23]. The downregulation of these three compounds suggests that ZEN elimination may suppress carbohydrate metabolism and energy consumption in lactic acid bacteria.

**Figure 7:**
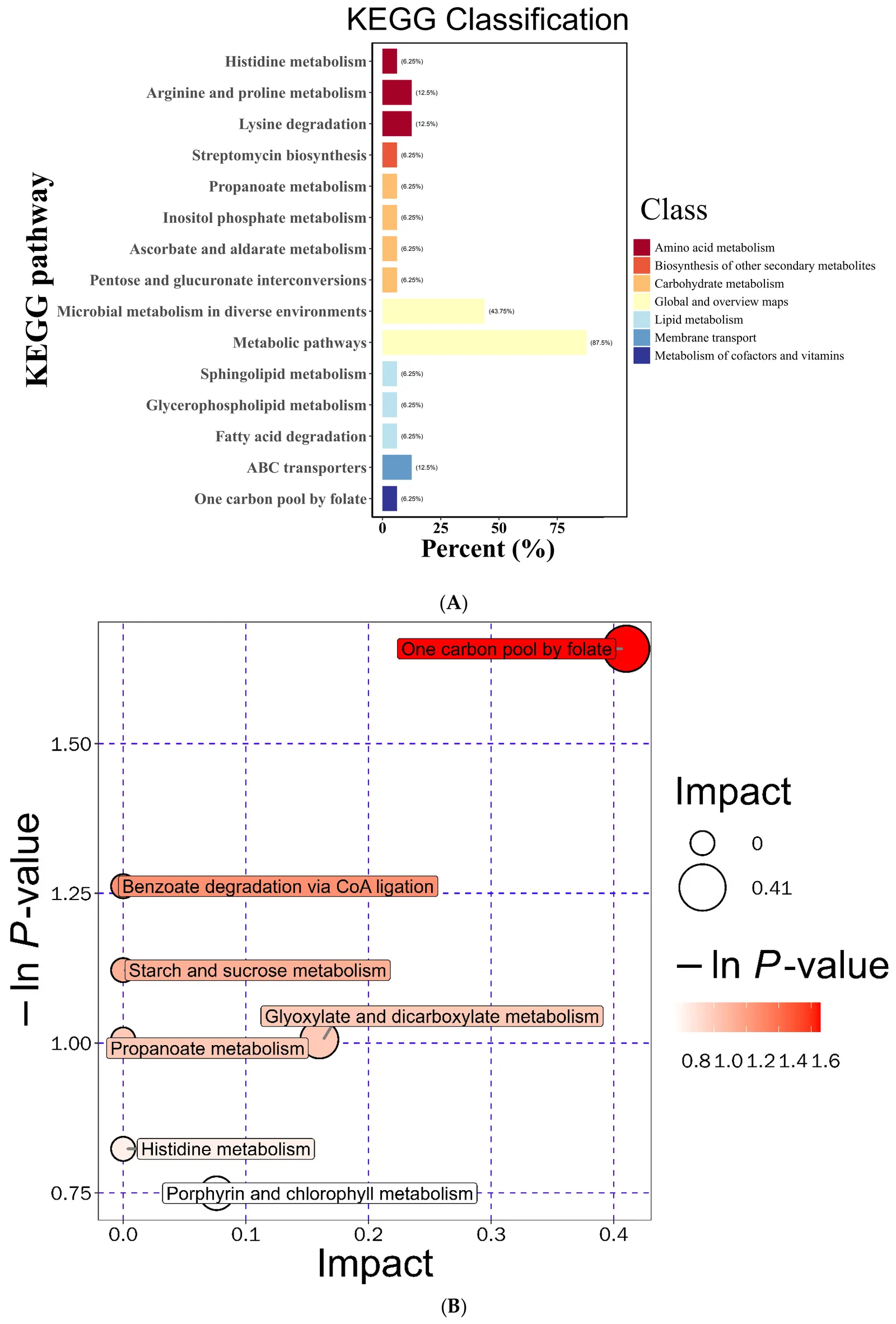
Kyoto Encyclopedia of Genes and Genomes (KEGG) classification chart of differential metabolites (**A**) and pathway analysis diagram (**B**).

A total of 357 differential metabolites were identified in *L. paracasei* 85 after ZEN treatment. Of these, 55 metabolites (15.4%) were successfully annotated and mapped to 28 metabolic pathways using the KEGG database. Regarding metabolite abundance, the primary enriched pathways included the general metabolic pathway, comprising 14 differential metabolites (seven upregulated and seven downregulated); microbial metabolism in diverse environments, with seven differential metabolites (five upregulated and two downregulated); and biosynthesis of secondary metabolites, containing six differential metabolites (four upregulated and two downregulated). Subsequently, seven key metabolic pathways potentially involved in ZEN removal by *L. paracasei* 85 were identified. As illustrated in Figure 7 and Table 2, the most significantly enriched pathway was the one-carbon pool by folate, which was mediated by 5,10-methenyltetrahydrofolic acid and glyoxylate and dicarboxylate metabolism. Reduced tetrahydrofolate, a family of enzyme cofactors, is converted to 5,10-methenyltetrahydrofolic acid via formaldehyde oxidation and plays a crucial role in various intracellular enzymatic reactions [24]. The glyoxylate and dicarboxylate metabolic pathways are intricately linked with carbohydrate metabolism, suggesting that intracellular energy metabolism is crucial for ZEN removal.

### 2.8. Combined Functional Enrichment Analysis of Transcriptome and Metabolome

Transcriptomic data provide a comprehensive overview of the dynamic regulation of gene expression, whereas metabolomic data capture global alterations in intracellular metabolite levels. By performing an integrated functional enrichment analysis of transcriptomic and metabolomic data, the relationships between differentially expressed genes and differential metabolites can be elucidated, thereby clarifying how changes in gene expression influence metabolite synthesis and degradation, and subsequently affect metabolic pathway activity. In this study, transcriptomic profiles were integrated with KEGG pathway annotations of differential metabolites, resulting in the identification of eight metabolites associated with carbohydrate metabolism. Heatmap analysis revealed that the levels of carbohydrate metabolism-related metabolites were generally lower in the experimental group than in the control group.

**Figure 8:**
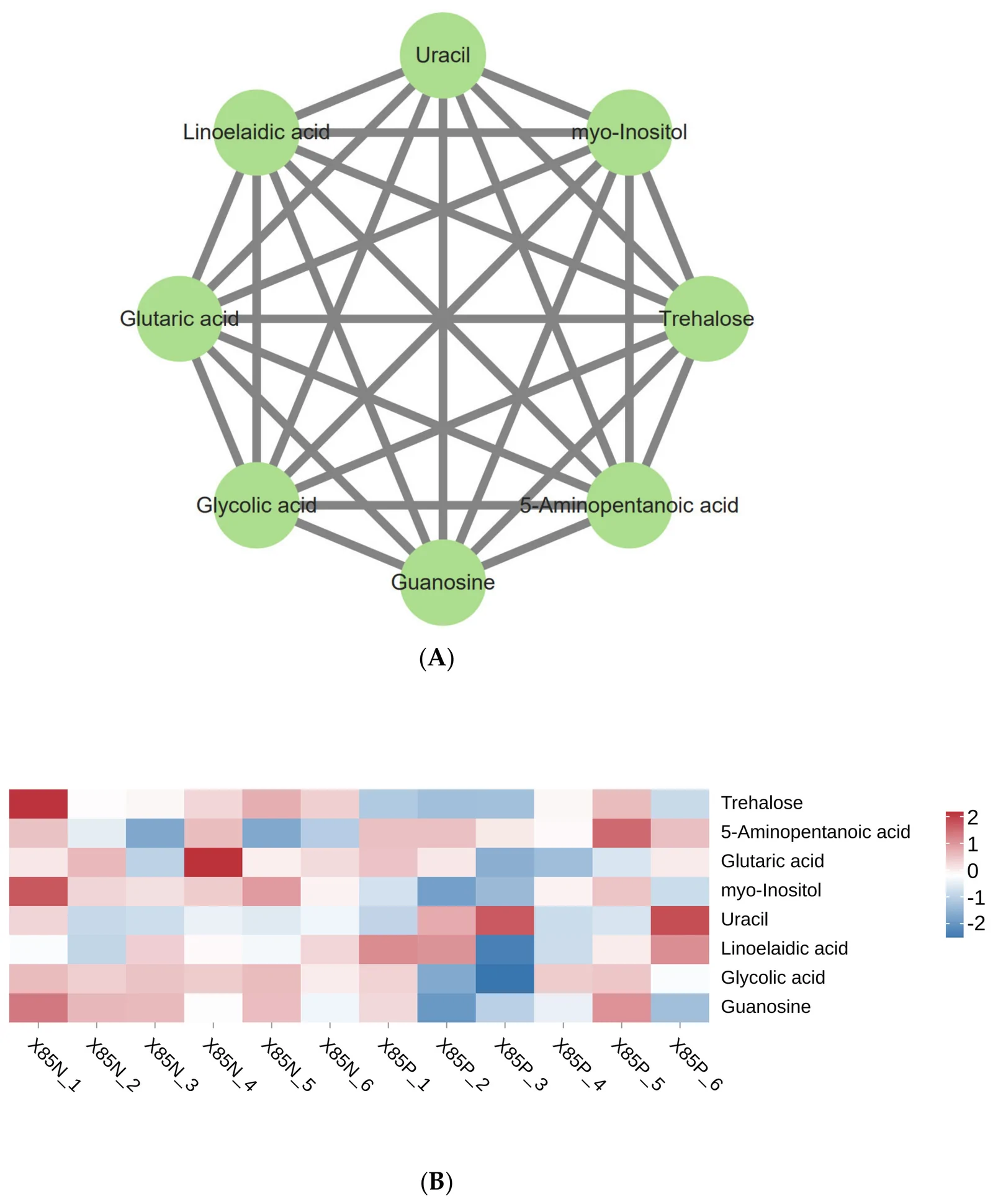
Network diagram of target metabolites in sugar metabolism (**A**) and analytical results for target metabolites (**B**).

These findings demonstrate that the presence of ZEN in the medium may suppress carbohydrate metabolism pathways, potentially disrupting the central carbon metabolism of the strain. Correlation analysis between carbohydrate metabolism-related genes and metabolites revealed significant associations with four key metabolites: myo-inositol, guanosine, glycolic acid, and trehalose (Figure 8). As key components of carbohydrate metabolism pathways, alterations in the abundance of these four metabolites and their correlations with related genes may contribute to the regulatory mechanisms underlying the observed downregulation of carbohydrate metabolism in the experimental group.

## 3. Discussion

Phenotypic verification confirmed that *L. paracasei* 85 exhibited a ZEN degradation efficiency of 78.4% under the current experimental conditions (5.0 mg/L ZEN, same culture medium, temperature and incubation time as those used in omics sample preparation), which validated the stable detoxification performance of this strain and guaranteed the authenticity of subsequent multi-omics analysis [18]. According to published studies, Lactobacillus have detoxification efficiencies that range from 30–55%, with *L. paracasei* strains exhibiting ZEN removal rates of 42–65% [25,26]. The majority of lactic acid bacteria eliminate ZEN via passive physical adsorption, which is highly susceptible to environmental interference [25,26,27,28]. The other LAB remove ZEN through the secretion of secondary metabolites or specific enzymes during microbial growth and metabolism, which convert toxins into less toxic or non-toxic degradation products under mild conditions [29].

Microbial sources play a pivotal role in contemporary research, particularly in mycotoxin degradation in food and feed systems. Microorganisms and enzymes derived from food systems are generally regarded as safe and reliable, making them an important focus of current research [30,31]. Recent studies have highlighted the potential of lactic acid bacteria as effective agents for mycotoxin removal, attributed to their symbiotic interactions within the intestinal environment and their ability to produce metabolites and enzymes such as esterases and organic acids [32,33,34]. Despite these advancements, most existing research has focused on screening strains and optimizing toxin removal efficiency, with limited exploration of the underlying molecular mechanisms [35,36].

ZEN exhibits significant genotoxic properties and can induce DNA strand breaks and chromosomal aberrations by inducing oxidative stress [37,38]. Folate-mediated one-carbon metabolism provides essential one-carbon units that are necessary for purine and thymine biosynthesis, thereby providing a crucial foundation for DNA replication and repair processes. Differential metabolites often exhibit similar or complementary biological functions or common metabolic pathways, resulting in analogous or contrasting expression patterns among different groups. The notable accumulation of 5,10-methenyltetrahydrofolate, coupled with the concurrent activation of replication and repair pathways at the transcriptomic level, indicates that *L. paracasei* 85 initiates nucleotide pool replenishment. This physiological adaptation facilitates the survival and effective biodegradation of ZEN under high ZEN concentrations. In summary, this study elucidated the metabolic response of *L. paracasei* 85 to ZEN at the multi-omics level, enhancing our understanding of the molecular mechanisms underlying ZEN degradation by lactic acid bacteria. These findings provide a theoretical basis and promising strain resource for the development of food-grade biological agents targeting mycotoxins, thereby contributing to the safety and assurance of cereals and related products.

Notably, according to the findings of our previous study, *L. paracasei* 85 has been confirmed to possess genuine ZEN biodegradation capacity based on the detection of degraded ZEN metabolites, rather than merely exhibiting passive stress responses to ZEN toxicity [18]. On this premise, the metabolic reprogramming observed in the present study comprehensively reflects two coordinated bacterial behaviors under ZEN exposure: active toxin detoxification metabolism and adaptive stress resistance. The upregulated lipid and amino acid catabolism, together with the reprogrammed carbohydrate and one-carbon metabolism, are not only simple stress responses to ZEN toxicity but also serve as core metabolic supports for strain survival and efficient ZEN biotransformation. This multi-omics evidence further explains the molecular basis of the stable and high ZEN removal efficiency of *L. paracasei* 85, which is significantly superior to the passive physical adsorption mechanism of most traditional lactic acid bacteria.

## 4. Conclusions

Transcriptomics and metabolomics have emerged as indispensable omics technologies, offering significant advantages in elucidating microbial detoxification processes and molecular response mechanism. These technologies provide a comprehensive understanding of toxin removal mechanisms, from gene regulation to the metabolic processes. They enable the precise identification of key functional genes, core metabolic pathways, and characteristic metabolites, thereby providing multidimensional and in-depth theoretical insights into the molecular mechanisms underlying the elimination of toxins.

Carbohydrate metabolism is a fundamental metabolic process in living organisms. Our findings indicate that most differentially expressed genes were significantly enriched in carbohydrate metabolism pathways, with the pentose phosphate pathway exhibiting notable enrichment. This suggests that ZEN disrupts the basal metabolic processes in this strain. Furthermore, the phosphoenolpyruvate-dependent sugar phosphotransferase system was identified as the most significantly enriched pathway, consistent with the metabolomic results. Metabolomic analysis revealed a substantial reduction in trehalose levels and an apparent increase in activity within the pentose and glucuronate interconversion pathways, suggesting that *L. paracasei* 85 may enhance extracellular carbohydrate uptake and carbon metabolic flux in response to ZEN stress. Folate-mediated one-carbon metabolism exhibited the highest pathway enrichment score.

This study presented ZEN removal-specific metabolic regulation and constructed a core gene–metabolite interaction network responsible for ZEN bioremoval in *L. paracasei* 85. These findings provide systematic molecular evidence for the unique detoxification characteristics of this strain, offering solid theoretical support for the functional optimization and industrial application of probiotic-based ZEN detoxification technology. This study’s findings imply a potential intrinsic linkage between carbohydrate metabolic fluctuation and ZEN biodegradation. It is speculated that remodeled carbohydrate metabolism provides essential energy and metabolic precursors to support bacterial stress tolerance and subsequent mycotoxin removal [17]. Future studies will focus on identifying these potential sugar-conjugated ZEN metabolites to further elaborate the precise regulatory mechanism linking carbohydrate metabolism and ZEN detoxification, contributing to the establishment of a molecular regulatory network for probiotic mycotoxin elimination.

## 5. Materials and Methods

### 5.1. Materials and Reagents

*L. paracasei* 85, previously isolated and identified in our laboratory [18], was used in this study. The ZEN standard was purchased from Anpel Laboratory Technologies (Shanghai, China). Chromatographic-grade methanol and acetonitrile were supplied by CNW Technologies GmbH (Shanghai, China). Chromatography-grade ammonium acetate was obtained from Sigma-Aldrich (St. Louis, MO, USA). Ammonia solution (chromatographic grade) was obtained from Thermo Fisher Scientific (Waltham, MA, USA). The MRS culture medium was purchased from Guangdong Huankai Microbial Science & Tech. Co., Ltd. (Guangzhou, China). The fungal Total RNA Rapid Extraction Kit was obtained from Sangon Biotech Co., Ltd. (Shanghai, China). The Reverse Transcription Kit was supplied by Vazyme Biotech Co., Ltd. (Nanjing, China).

### 5.2. Instruments and Equipment

High-performance liquid chromatography-tandem mass spectrometry, refrigerated centrifuges, and vortex mixers were supplied by Thermo Fisher Scientific (Waltham, MA, USA). The analytical balance and tissue grinders were purchased from Shanghai Jingxin Technology Co., Ltd. (Shanghai China). An ultrasonic instrument was obtained from Shenzhen Leidebang Electronics Co., Ltd. (Shenzhen, China). The Class II Biological Safety Cabinet (Model 1348) was provided by Sujie Medical Devices Co., Ltd. (Suzhou, China). An electric constant-temperature blast-drying oven was purchased from Shanghai Kuntian Laboratory Instruments Co., Ltd. (Shanghai, China). The real-time fluorescent quantitative PCR system was manufactured by Applied Biosystems (Foster City, CA, USA).

### 5.3. Methods

#### 5.3.1. Sample Pretreatment

Sample pretreatment method has been cited in previous publication [18]. The strain was subcultured three consecutive times in MRS broth, and the bacterial suspension was adjusted to a concentration of 1 × 10^6^ CFU/mL. The suspension was inoculated at a volume ratio of 2% (*v*/*v*) into MRS medium containing ZEN at a final concentration of 5.0 μg/mL, which served as the experimental group. MRS medium devoid of ZEN was used as the control. All samples were incubated at 37 °C and 180 rpm for 24 h. Subsequently, the cultures were centrifuged at 12,000 rpm for 10 min in a refrigerated centrifuge. The supernatant was discarded, and the cell pellets were rinsed three times with pre-cooled sterile phosphate-buffered saline for subsequent use. Three biological replicates were used in this experiment. The experimental group was designated as 85P, whereas the control group was designated as 85N.

#### 5.3.2. Transcriptome Sequencing and Data Analysis of *L. paracasei* 85

The rinsed samples were preserved in liquid nitrogen and sent to Majorbio Bio-Pharm Technology Co., Ltd. (Shanghai, China) for transcriptome sequencing and analysis. Total RNA and mRNA samples were used for library construction and sequencing, respectively. The raw sequencing data were filtered to obtain clean reads. A reference genome index was constructed, and clean reads were aligned to the reference genome to generate mapped reads for further analysis. Differential expression analysis was conducted using DESeq2 (v1.52.0) in R (v4.6.0) and a negative binomial distribution model. Genes with an absolute ∣log_2_ (fold change)∣ > 1.2 and a *p*-value < 0.05 were defined as significantly differentially expressed and were included in subsequent analyses.

#### 5.3.3. Validation Using RT-qPCR

Using NR_025880.1 as the reference gene, seven key differentially expressed genes closely linked to carbohydrate metabolism were selected for RT-qPCR to validate the reliability of the transcriptome data. Specific primers were designed using Primer 5.0 (Table 3) and synthesized by Sangon Biotech Co., Ltd. (Shanghai, China). Total RNA was extracted from the bacterial strains using a bacterial total RNA extraction kit according to the manufacturer’s protocol. Subsequently, RNA was reverse-transcribed into cDNA using a reverse transcription kit for relative quantitative analysis. The PCR system, in a total volume of 20 μL, comprised 10 μL of SYBR qPCR Master Mix, 4 μL of cDNA template, 0.4 μL of each forward and reverse primer, and 5.2 μL of deionized water. The thermal cycling conditions were as follows: initial denaturation at 95 °C for 90 s; followed by 40 cycles of denaturation at 95 °C for 5 s, annealing at 60 °C for 15 s, and extension at 72 °C for 20 s. The resulting data were analyzed using the 2^−ΔΔCt^ method.

#### 5.3.4. Metabolome Sequencing and Analysis of *L. paracasei* 85

The prepared samples were shipped on dry ice to BioTree Biotechnology Co., Ltd. (Shanghai, China) for metabolomic analysis. Upon arrival, the samples were thawed in an ice bath, and 2 mL of double-distilled water was added. The samples were homogenized at 35 Hz for 4 min and subjected to ultrasonication for 5 min in an ice bath. The extraction process was repeated three times. Finally, 200 μL of the homogenate was mixed with 800 μL of an extraction solution consisting of methanol and acetonitrile (1:1, *v*/*v*) containing an isotopically labeled internal standard mixture. The mixture was ultrasonicated for 10 min in an ice bath and incubated at −40 °C for 1 h. Subsequently, the samples were centrifuged at 12,000 rpm and 4 °C for 15 min, after which the supernatant was transferred to sample vials for further analysis.

Chromatographic separation was performed using a Vanquish ultra-high-performance liquid chromatography system (Thermo Fisher Scientific, Waltham, MA, USA) equipped with a Waters ACQUITY UPLC BEH Amide column (2.1 mm × 100 mm, 1.7 μm; Waters Corporation, Milford, MA, USA). Mobile phase A consisted of an aqueous solution containing 25 mM ammonium acetate and 25 mM aqueous ammonia, and mobile phase B consisted of acetonitrile. The sample tray temperature was maintained at 4 °C with an injection volume of 2.0 μL. The experiment included six biological replicates.

The raw data were converted to mzXML format using ProteoWizard. Peak identification, extraction, alignment, and integration were performed using R packages. Metabolite annotation was conducted by matching the data against the in-house secondary mass spectrometry database BiotreeDB (V2.1), with the algorithm cutoff value set at 0.3. Data analysis was performed following the data preprocessing.

## Figures and Tables

**Table 1:** Quality control data statistics of transcriptome sequence.

Sample	Raw Reads (G)	Clean Reads (G) ^a^	Clean Q20 (%) ^b^	Clean Q30 (%) ^c^	Genome Mapped Reads (G) ^d^	Genome Mapped Ratio (%) ^e^
85N_1	3.11	3.02	98.21	94.96	3.01	93.35
85N_2	3.00	2.90	98.19	94.95	2.73	93.96
85N_3	3.33	3.23	98.24	95.09	2.83	93.79
85P_1	3.22	3.14	98.25	95.12	2.79	93.30
85P_2	2.94	2.86	98.24	95.07	2.69	93.83
85P_3	3.12	2.99	98.27	95.18	2.95	94.04

^a^: Number of clean reads obtained after filtering raw reads; ^b^,^c^: percentage of bases with a Phred score > 20 and Phred score > 30 among the total bases, respectively; ^d^: number of reads mapped to the reference genome; ^e^: percentage of mapped reads relative to clean reads.

**Table 2:** Metabolic pathway analysis of *L. paracasei* 85 following ZEN treatment.

Pathway Name	Number of Metabolites	Number of Differential Metabolites	Differential Metabolite Name
One carbon pool by folate	7	1	5,10-Methenyltetrahydrofolic acid (cpd:C00445)
Benzoate degradation via CoA ligation	11	1	Benzoic acid (cpd:C00180)
Starch and sucrose metabolism	13	1	Trehalose (cpd:C01083)
Propanoate metabolism	15	1	2-Hydroxybutyric acid (cpd:C05984)
Glyoxylate and dicarboxylate metabolism	15	1	5,10-Methenyltetrahydrofolic acid (cpd:C00445)
Histidine metabolism	19	1	Imidazole-4-acetaldehyde (cpd:C05130)
Porphyrin and chlorophyll metabolism	21	1	Porphobilinogen (cpd:C00931)

**Table 3:** Primers used in this study.

Primer Name	Forward Primer Sequence (5′-3′)	Reverse Primer Sequence (3′-5′)
GWC93_RS09170	GACACGGACCGGATTATTG	TAAGCATAGCTGGCTTCATTAT
GWC93_RS09165	TATCGGTCCTGATGGGATAG	CGAATGAAGGAGCCTAAGTG
GWC93_RS04540	CCTCATGCTGATCGTGTATTA	CAGCCTGGAGTATATGGAATG
GWC93_RS00680	CGGATACCGTCATTGCTATC	TGAACCAGTCCCAGAAGT
GWC93_RS05825	CTTATCGTCATCGGCTTTATCT	AGTCCTAAACCAACTGGAATG
GWC93_RS03160	GGCTTCGGCAGCTTATTAT	CAATGTTCTTGAGGTCTGTCT
GWC93_RS01280	GTCTCTGCTTCTGGAGTTTC	ACATAGAAATGGGCGTCATAG

## Data Availability

The data presented in this study are openly available in [National Microbiology Data Center (NMDC] at [https://nmdc.cn/resource/genomics/miscellan/detail/NMDCM0002660, accessed on 27 August 2026], reference number [NMDCM0002660, NMDCM0002661].

## Abbreviations

The following abbreviations are used in this manuscript:

ZEN	Zearalenone
EU	European Union
GO	Gene Ontology
MF	Molecular Function
CC	Cellular Component
BP	Biological Process
PTS	phosphotransferase system
RT-qPCR	Real-time Quantitative PCR
KEGG	Kyoto Encyclopedia of Genes and Genomes
